# Flavonoids Identification and Pancreatic Beta-Cell Protective Effect of Lotus Seedpod

**DOI:** 10.3390/antiox9080658

**Published:** 2020-07-24

**Authors:** Ming-Shih Lee, Charng-Cherng Chyau, Chi-Ping Wang, Ting-Hsuan Wang, Jing-Hsien Chen, Hui-Hsuan Lin

**Affiliations:** 1 Department of Medical Laboratory and Biotechnology, Chung Shan Medical University, Taichung City 40201, Taiwan; jdzlee@gmail.com (M.-S.L.); cshb015@csh.org.tw (C.-P.W.); benny791119@livemail.tw (T.-H.W.); 2 Department of Clinical Laboratory, Chung Shan Medical University Hospital, Taichung City 40201, Taiwan; 3 Research Institute of Biotechnology, Hungkuang University, Taichung City 43302, Taiwan; ccchyau@sunrise.hk.edu.tw; 4 Department of Nutrition, Chung Shan Medical University, Taichung City 40201, Taiwan

**Keywords:** oxidative stress, pancreatic beta-cell, lotus seedpod, apoptosis, autophagy

## Abstract

Oxidative stress is highly associated with the development of diabetes mellitus (DM), especially pancreatic beta-cell injury. Flavonoids derived from plants have caused important attention in the prevention or treatment of DM. Lotus seedpod belongs to a traditional Chinese herbal medicine and has been indicated to possess antioxidant, anti-age, anti-glycative, and hepatoprotective activities. The purpose of this study was to demonstrate the pancreatic beta-cell protective effects of lotus seedpod aqueous extracts (LSE) against oxidative injury. According to HPLC/ESI-MS-MS method, LSE was confirmed to have flavonoids derivatives, especially quercetin-3-glucuronide (Q3G). In vitro, LSE dose-dependently improved the survival and function of rat pancreatic beta-cells (RIN-m5F) from hydrogen peroxide (H_2_O_2_)-mediated loss of cell viability, impairment of insulin secretion, and promotion of oxidative stress. LSE showed potential in decreasing the H_2_O_2_-induced occurrence of apoptosis. In addition, H_2_O_2_-triggered acidic vesicular organelle formation and microtubule-associated protein light chain 3 (LC3)-II upregulation, markers of autophagy, were increased by LSE. Molecular data explored that antiapoptotic and autophagic effects of LSE, comparable to that of Q3G, might receptively be mediated via phospho-Bcl-2-associated death promoter (p-Bad)/B-cell lymphoma 2 (Bcl-2) and class III phosphatidylinositol-3 kinase (PI3K)/LC3-II signal pathway. In vivo, LSE improved the DM symptoms and pancreatic cell injury better than metformin, a drug that is routinely prescribed to treat DM. These data implied that LSE induces the autophagic signaling, leading to protect beta-cells from oxidative stress-related apoptosis and injury.

## 1. Introduction

Diabetes mellitus (DM) is a chronic and complex illness characterized mainly by hyperglycemia and other metabolic disorders with several symptoms, including polyphagia, polydipsia, polyuria, and pancreatic β-cell (beta-cell) mass loss [1,2]. In hyperglycemia condition, insulin resistance, cell inflammation, and excess of lipids and other metabolic fuels, containing fatty acid and glucose, trigger beta-cell dysfunction over time [1,3]. It has been further indicated that hyperglycemia-induced reactive oxygen species (ROS) generation causes beta-cell dysfunction, playing a critical role in the DM progression [4]. A decrease in beta-cell number caused by increased apoptosis and impaired insulin production and secretion from the remaining beta-cells lead to beta-cell dysfunction [1,3]. In DM, hyperglycemia has been shown to cause apoptosis of beta-cells via the intrinsic pathway involving the molecular factors of B-cell lymphoma 2 (Bcl-2) protein family [5]. Thus, preventing apoptosis of beta-cells could be a valuable strategy to manage or treat DM, actually reversing and inhibiting this illness to an advanced degree instead of just lowering blood glucose.

Autophagy has been shown to associate with the lysosomal pathway, which removes damaged or excess organelles, thereby maintaining cellular homeostasis and regulating cell growth and metabolism [6]. Previous studies have reported that autophagy, which leads to many cellular physiological adaptations, is closely associated with the homeostasis of beta-cells [7,8]. Autophagy not only protects beta-cells from injury and apoptotic program but also maintains the beta-cell mass, number, and insulin-secreting function [9]. Nutrient overload leads to defective autophagy, resulting in accumulations of autophagy substrates like p62 and autophagosomes, lysosomal dysfunction, and inactivation of multiprotein complexes, thereby contributing to beta-cell injury [10]. Among them, class III PI3K (phosphatidylinositol-3 kinase)/the mammalian homolog of autophagy-related genes (Atg) 6, also known as Beclin-1, an assembly of the lipid kinase complex, initiates nucleation of the isolation membrane (pre-autophagosomal membrane). There are other two ubiquitin-like conjugation signaling pathways that stimulate the pre-autophagosomal membrane expansion, containing mainly microtubule-associated protein light chain 3 (LC3)-II and Atg5/12 conjugate [11]. 

Lotus seedpod, the mature torus of Nelumbo nucifera Gaertn., is used as a traditional Chinese herbal medicine for eliminating bruise and with hemostasis function [12]. Previous studies have demonstrated that lotus seedpod is rich in procyanidins [12,13], and oligomeric procyanidins of lotus seedpod (LSOPC) have been further extracted and shown to have multiple biological activities, including antioxidant, anti-age, anti-cancer, and anti-glycative effects [14,15,16,17,18,19]. In the literature, LSOPC scavenged reactive carbonyls and reduced advanced glycation end-products (AGEs) formation [17] and alleviated the symptoms and progression of nonalcoholic fatty liver disease (NAFLD) in a high-fat diet (HFD)-induced obesity rat model [18], as well as enhanced glucose homeostasis in a DM model of streptozotocin (STZ)-injected mice [19]. Our recent studies have also reported the hepatoprotective activity of lotus seedpod aqueous extracts (LSE) against an unsaturated fatty acid oleic acid (OA) and endotoxin lipopolysaccharide (LPS)-induced liver injury [20,21]. However, there are limited reports on the detailed characterization of LSE. In this study, the compositions of LSE were identified by using high-performance liquid chromatography (HPLC)/electrospray ionization tandem mass spectrometry (ESI-MS-MS) analysis, and the ability of LSE to protect beta-cells against oxidative injury in vitro and in vivo models was evaluated.

The beta-cells have low content in antioxidant enzymes and are more sensitive to oxidative stress than other types of cell lines [22]. It has been reported that free radical scavengers or antioxidants in pancreatic tissues or islets can protect beta-cells from oxidative stress [23]. In the text, the purpose of the study was to explore the protective effects of LSE on beta-cells from oxidative injury. Hydrogen peroxide (H_2_O_2_) as a direct oxidant and LSE were administered to beta-cells, cellular growth and apoptosis were examined, and the molecular mechanism(s) underlying the autophagic induction by LSE were also evaluated. These effects of pre-treatments of autophagy inhibitors—3-methyladenine (3-MA) and chloroquine (CQ)—on the beta-cell protective potential of LSE were further investigated. In an HFD combined with the STZ-induced diabetic mice model, the effects of LSE on serum parameters, glucose tolerance, pancreatic tissue, and protein expressions of the apoptotic and autophagic factors were clarified.

## 2. Materials and Methods

### 2.1. Preparation, Total Flavonoids Content, and HPLC/ESI-MS-MS Analysis of LSE

Raw lotus seeds (cultivar: Sheklian), purchased from Baihe District, Tainan City, Taiwan, ROC, were dried in an oven at 40 °C for one week at least and cooled by air at ambient temperature. All dried plant samples were then stored in a dry cool place before a further extract of LSE. The dried lotus seedpods (150 g) were boiled with 95 °C hot water (6 L). After 2 h, the aqueous extract was then evaporated under vacuum at −80 °C. In a final process, the extracted solution was filtered and lyophilized to gain a powder, which was the aqueous fraction of LSE. The yield rate of LSE is approximately 27%, and it is stored at −80 °C before experimental use. The total flavonoids content of LSE was analyzed as rutin equivalents utilizing a modified colorimetric assay [24] and represented as % (*w*/*w*) of the aqueous extract.

The HPLC/ESI-MS-MS method for LSE was carried out using the Waters Symmetry (Waters Corp., Milford, MA, USA) column (3.5 µm, 2.1 × 150 mm) fitted with a Security-Guard Ultra C18 sub-2-µm particle column with 2.1 mm × 2.0 mm (Phenomenex, Inc., Torrance, CA, USA) utilizing an HPLC system with diode array detector (DAD). The column temperature was maintained at 35 °C. The elution solvent system was carried out, and the flow rate was fixed at 0.3 mL/min during the elution process, with gradient elution utilizing two solvents, including solvent A (water containing 0.1% formic acid) and solvent B (acetonitrile containing 0.1% formic acid). The gradient elution was performed with isocratic elution at 5% B for 5 min, 5–35% B in 5–25 min, 35–60% B in 25–30 min, 60–95% B in 30–40 min, and last 95% B isocratic for 15 min. Next, the absorption spectra of eluted compounds were scanned within 210 to 600 nm, utilizing the in-line DAD monitored at 254, 280, 325, and 375 nm, respectively. By a triple quadruple mass spectrometer, the active compounds having been separated and eluted were further identified. Subsequently, the system was, respectively, operated, utilizing ESI with positive and negative ionization modes in the potential of both + and −3700 V applied to the capillary tip. Using an autosampler, sample extracts (5 μL) were directly injected into the column. Nitrogen was utilized as not only a collision gas but also the drying gas at a flow rate of 9 L/min, the drying gas temperature was set at 300 °C, and the nebulizing gas was maintained at a pressure of 40 psi. The in-source collision-induced dissociation (CID) voltage was 15 V, while the fragmentor voltage was 85 V. Quadrupole 1 filtered the calculated *m*/*z* of each interest compound, and at a scan time of 200 ms/cycle, quadrupole 2 scanned for ions generated by nitrogen collision between the ionized compounds in the range of 100–800 amu. By comparing their mass spectra provided from ESI-MS and ESI-MS/MS with those of authentic standards, the identification of separated compounds in LSE was performed. 

### 2.2. Cell Culture

The rat pancreatic beta-cell line (RIN-m5F), obtained from the Bioresource Collection and Research Center (Food Industry Research and Development Institute, Hsinchu City, Taiwan, ROC), was cultured in RPMI-1640 medium supplemented with 10% fetal bovine serum (FBS, Thermo Fisher Scientific, Inc., Waltham, MA, USA) and 1% penicillin-streptomycin (Gibco/BRL, Gaithersburg, MD, USA). Cell cultures were placed and maintained at 37 °C in a humidified atmosphere with 5% CO_2_ and passaged by trypsinization every three days. The cells (passage: 45–70) were subcultured under the conditions indicated for each experiment.

### 2.3. Cytotoxicity Analysis

#### 2.3.1. 3-(4,5-Dimethyl-2-Thiazolyl)-2,5-Diphenyl-2H-Tetrazolium Bromide (MTT) Method 

In order to determine the inhibitory effect of LSE against H_2_O_2_-induced cytotoxicity, the MTT method was carried out, as described previously [25]. RIN-m5F cells were planted at the denseness of 10^5^ cells/mL and treated with or without H_2_O_2_ or/and LSE at 0.5 and 1 μg/mL for 24 h. Thereafter, the culture medium was replaced, and MTT solution (0.1 mg/mL), purchased from Sigma Chemical Co., (St. Louis, MO, USA), was then added for the 4-h incubation. Following the solubilization, the analysis was performed with isopropanol via spectrophotometer at 563 nm, and the viable cell number was directly proportional to the formazan production. The concentration of H_2_O_2_ on the inhibition of 60 percent (IC_60_) of RIN-m5F cell survival was about 200 μM. Therefore, H_2_O_2_ at 200 μM for 24 h was selected as a further cellular oxidative injury model. The MTT assay was also performed to determine the effect of the test LSE (0–100 μg/mL) alone on RIN-m5F cell growth and to further evaluate the non-cytotoxic concentrations [20].

#### 2.3.2. Glucose-Stimulated Insulin Secretion (GSIS) Assay

To evaluate the insulin-secreting effect of LSE on the H_2_O_2_-treated cells, RIN-m5F cells at the denseness of 10^5^ cells/mL were plated in 24-well plates and treated with or without LSE (0.5 and 1.0 μg/mL) in the presence of H_2_O_2_ (200 μM). After 24 h, the treated cells were placed in glucose-free Krebs–Ringer bicarbonate (KRB) solution, containing 4.7 mmol/L KCl, 115 mmol/L NaCl, 1.2 mmol/L KH_2_PO_4_, 1.2 mmol/L MgSO_4_, 20 mmol/L NaHCO_3_, 16 mmol/L HEPES, 2.56 mmol/L CaCl_2_, and 0.2% bovine serum albumin (BSA), and the cells were handled in KRB solution with low dose (3.3 mM) or high dose (16.7 mM) of glucose for 1 h. After incubation for 1 h at 37 °C, the supernatant was then collected, and the content of insulin was detected by enzyme-linked immunosorbent assay (ELISA) (Mercodia AB, Uppsala, Sweden)

#### 2.3.3. Lipid Peroxidation Assay

By evaluating thiobarbituric acid relative substances (TBARS, nmol/mg protein) via fluorescence spectrophotometer at an excitation (532 nm) and emission (600 nm) wavelength, respectively, the cellular level of lipid peroxidation was analyzed [26]. Quantification of intracellular TBARS level was further determined by comparison with a standard curve of the lipid peroxidation product malondialdehyde (MDA) equivalents produced using acid-catalyzed hydrolysis of 1,1,3,3-tetramethoxypropane (Sigma Chemical, Co., St. Louis, MO, USA).

#### 2.3.4. Reactive Oxygen Species (ROS) Content Analysis

To study the influence of LSE on intracellular ROS content upon H_2_O_2_ stimulation, dichlorofluorescein diacetate (DCFH-DA), as a fluorescent probe, obtained from Enzo Life Sciences Inc. (Farmingdale, NY, USA), was utilized. The confluent RIN-m5F cells at the denseness of 10^5^ cells/mL were plated in 6-well plates and treated with or without LSE (0.5 and 1 μg/mL) in the presence of H_2_O_2_ (200 μM). After 24 h, the treated medium was removed, and then the cells were treated with 2 µM DCFH-DA at 37 °C for the next 30 min. Utilizing Muse™ Cell Analyzer (EMD Millipore Corporation, Merck Life Sciences, KGaA, Darmstadt, Germany), the fluorescence intensity of ROS generation was measured. The values in each group were represented relative to the fluorescence signal of the untreated control.

### 2.4. Apoptosis Analysis

#### 2.4.1. Cell Cycle Distribution Analysis

Using a flow cytometry (Becton Dickinson, San Jose, CA, USA) method, the quantification of cell cycle distribution was studied. In brief, after the treated cells were rinsed twice with phosphate-buffered saline (PBS), the cell suspension was centrifuged at 1500× *g* rpm for 5 min at room temperature. All the supernatant was decanted, followed by adding 70% methanol (1 mL) to the pellet at −20 °C for 24 h at least. Afterward, the mixture was stained with cold propidium iodide (PI, 1 mL) reagent, containing 20 μg/mL PI, 20 μg/mL RNase A, and 0.1% Triton X-100 (all chemicals from Sigma-Aldrich, St Louis, MO, USA), for 15 min at room temperature in the dark, prior to the samples being measured using a FACScan flow cytometer. The PI was excited at 488 nm, and the fluorescence signal was subjected to logarithmic amplification with red fluorescence from PI being detected above 600 nm. Each phase of the cell cycle was expressed as the cell number versus the DNA amount, as indicated by fluorescence intensity, and gated into four phases, including subG1 (hypodiploid cells), G0/G1, S, and G2/M with CELLQuest Version 3.3 software. The % of cells in each phase over total cells was also measured.

#### 2.4.2. 4′,6-Diamidino-2-Phenylindole Dihydrochloride (DAPI) Staining

By analyzing the fluorescence microscopy of DAPI-stained cells, the apoptotic morphology characteristics were evaluated. The confluent RIN-m5F cells at the denseness of 10^5^ cells/mL were plated in 6-well plates and treated with or without LSE (0.5 and 1 μg/mL) in the presence of H_2_O_2_ (200 μM). After 24 h, the monolayer of cells was washed twice with PBS and then fixed with 4% paraformaldehyde for 30 min at room temperature. For the next 30 min, the fixed cells were stained with DAPI solution (1 μg/mL), purchased from Sigma Chemical Co., (St. Louis, MO, USA), followed by rinsing twice with PBS. Under 400× magnification using a fluorescent microscope with a 340/380 nm excitation filter, the nuclei of apoptotic cells, including intensely stained, condensed chromatin, and fragmented nuclei, were determined. The % of apoptosis was calculated and expressed as the ratio of DAPI-positive cells to total cells counted.

#### 2.4.3. Annexin V-Fluorescein Isothiocyanate (FITC) Staining

In order to quantify apoptotic cells, Annexin V-FITC detects the translocation of phosphatidylinositol from the inner to the outer cell membrane during early apoptosis, while 7-amino-actinomycin (7-AAD) can enter the cell in late apoptosis or necrosis [27]. The treated cells were rinsed in cold PBS and then re-suspended in 1× binding buffer. The above solution (100 μL) was then transferred to 5 mL culture tubes. Subsequently, the cells were incubated with annexin V-FITC (5 μL) and 7-AAD (10 μL), purchased from BD Bioscience (Franklin Lakes, NJ, USA), and gently mixed for 15 min at room temperature in darkness. The 1× binding buffer (400 μL) was then added to each tube and analyzed using a FACScan flow cytometer, following which 18,000 cells were counted at least for each measurement.

#### 2.4.4. Western Blotting

Western blot analysis was carried out in accordance with a previously reported procedure [20]. After treatments, the whole-cell lysate was mixed with RIPA buffer, including 0.1% sodium dodecyl sulfate (SDS), 0.5% deoxycholic acid, 1% NP-40, 50 mM Tris-base saline (TBS), and 150 mM NaCl (pH value 7.5), and extracted by utilizing sonication. Equal protein amounts were separated by 8–15% SDS-polyacrylamide gels and then transferred to nitrocellulose membranes. For blocking the non-specific binding, 5% nonfat dry milk was incubated with the membranes for 1 h at 4 °C and then overnight at 4 °C with the primary antibodies. The following primary antibodies were applied: against caspase-3 (cysteine-aspartic protease-3), PARP-1 [poly (ADP-ribose) polymerase 1], Bcl-2, Bax (Bcl-2-associated X protein), p-Bad (phospho-Bcl-2-associated death promoter), Bad (above antibodies from Santa Cruz Biotech, CA, USA), β-actin (Sigma Chemical Co., St. Louis, MO, USA), LC3-I/II, Atg5/12 conjugate, p62, class III PI3K, and Beclin-1 (Novus Biological Inc., Arapahoe County, CO, USA). After 24 h, the nitrocellulose membranes were further hatched with the horseradish peroxidase-conjugated secondary antibodies, obtained from Sigma Chemical Co. The detection of protein expressions was carried out by utilizing enhanced chemiluminescence (ECL) reagent from Amersham (Arlington Heights, IL, USA).

### 2.5. Autophagy Analysis

#### 2.5.1. Acridine Orange (AO) Staining

To study the effect of LSE on autophagy in the H_2_O_2_-treated beta cells, the volume of the cellular acidic compartment, an autophagy marker, was evaluated by staining with lysosomotropic agent AO (Sigma Chemical, Co., St. Louis, MO, USA). Therefore, AO was observed to move freely across the cell membrane and accumulated in an acidic compartment, where it was detected as fluorescence bright red. In brief, the treated cells were incubated with 1 μg/mL of AO for 15 min at room temperature in darkness. Acidic vesicular organelles were then detected and photographed with a fluorescent microscope.

#### 2.5.2. LC3 Immunofluorescence

LC3B-II immunofluorescence analysis was carried out using a flow cytometry method, as described previously [27]. The treated cells were fixed in 4% paraformaldehyde for 30 min at room temperature and blocked in 0.01% Triton X-100, 1× PBS, and 5% goat serum. Thereafter, the fixed cells were hatched with an anti-LC3B antibody overnight at 4 °C, washed twice with PBS, and then reacted with Alexa Fluor 488 goat anti-rabbit IgG (Zhongshan Biological Technology, Zhongshan, China) for 1 h at room temperature. The cells were further harvested, rinsed in PBS 3 times, and re-suspended in PBS (500 μL). Using a flow cytometry method, the detection of 1 × 10^5^ cells per sample was carried out. The data of viable cell counts were plotted and presented as fluorescence intensity.

### 2.6. Evaluation of DM Symptoms In Vivo

The animal experiment procedure is illustrated in Figure S1 and was approved by the Chung Shan Medical University animal care committee in accordance with the guidelines of the Institutional Animal Care and Use Committee (IACUC approval number: 1256). Male BALB/c mice weighing between 20 and 22 g were randomly divided into five experimental groups (*n* = 10 for each group) as follows: group I, negative control (NC, normal diet); group II, combination of HFD and low dose STZ (HFD/STZ); group III, HFD/STZ + 1% LSE (with LSE at 1% added); group IV, HFD/STZ + 2% LSE (with LSE at 2% added); group V, HFD/STZ + 300 mg/kg of metformin. The mice of group II-V were fed for 12 weeks on an HFD containing 89.8% standard Purina Chow (Purina Mills, Inc., St. Louis, MO, USA), 10% coconut oil, and 0.8% cholesterol to be effectively used to generate an animal model that mimic the natural history and metabolic characteristics of the common type 2 DM in humans [28]. To induce type 2 DM, as described in previous studies [28,29], the mice were treated with STZ (35 mg/kg in citrate buffer, pH 4.5) intraperitoneally (i.p.) for 5 consecutive days, while the mice of group I received the same volume of citrate buffer. The plasma glucose level was monitored on days 3 and 7 by enzymatic colorimetric methods using an automatic analyzer (Olympus AU2700, Olympus Co., Tokyo, Japan). Seven days after STZ injection, mice with fasting-blood glucose >216 mg/dL were considered diabetic [30] and then randomly divided into three groups. At the same time, groups III and IV were further treated with oral feeding of LSE at 1 or 2 g mixed with 99 or 98 g normal diet, whereas the metformin group was administered intragastrically (i.g.) a metformin hydrochloride water solution (300 mg/kg) [31]. After 6 weeks of supplementation, oral glucose tolerance test (OGTT) was carried out on the day before the sacrifice. All mice were given glucose solution at 2 g/kg orally, and samples of blood from each animal were obtained at distinct intervals of time 30, 60, 90, and 120 min and quantified for plasma glucose. At the time of sacrifice, the blood of mice was collected, and then the levels of serum lipids, glucose, insulin, and other variables were measured by an automatic biochemical analyzer (Hitachi AutoAnalyzer 7020, Hitachi Co., Ltd., Tokyo, Japan). The homeostasis model of insulin resistance (HOMA-IR) was further calculated by utilizing glucose and insulin measurements as follows: HOMA-IR = fasting insulin μU/mL × fasting glucose (mmol/L)/22.5 [32]. Pancreatic tissues were rapidly dissected out and kept at −80 °C or in 10% neutral buffered formalin. For the histologic examination, the paraffin-embedded tissue sections from the pancreas were then stained with hematoxylin and eosin (H&E). TUNEL (terminal deoxynucleotide transferase dUTP nick end labeling) assay was performed for labeling DNA breaks to detect apoptotic cells by immunohistochemistry (IHC), as previously described [33]. The pancreatic tissue homogenates were centrifuged (10,000× *g* for 20 min) at 4 °C, and the supernatants of whole tissue extracts were used for TBARS, H_2_O_2_ level, measured by H_2_O_2_ assay kits (BioVision Incorporated, Milpitas, CA, USA), and Western blotting.

### 2.7. Statistical Analysis

Data are reported as means ± standard deviation (SD) of three independent experiments and evaluated by one-way analysis of variance (ANOVA). Significant differences were established at *p* < 0.05.

## 3. Results

### 3.1. LSE Is Rich in Flavonoids

**Figure 1:**
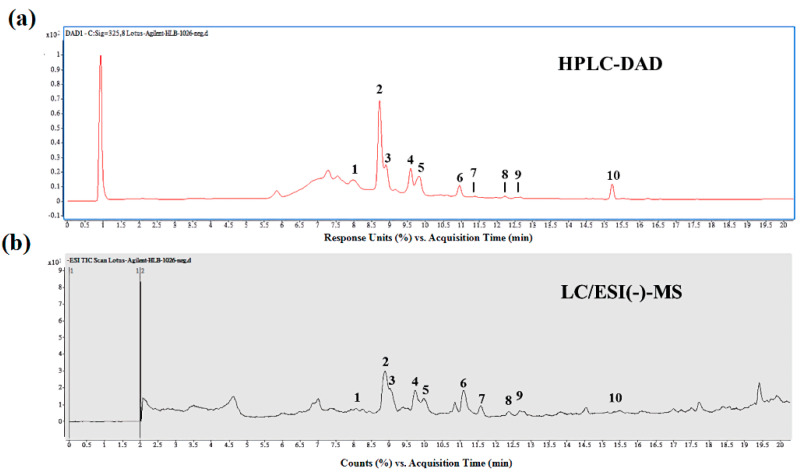
Component identification of lotus seedpod aqueous extracts (LSE). HPLC-DAD (high-performance liquid chromatography-diode array detector) chromatogram at 325 nm (**a**) and LC–ESI (−)-MS total ion current chromatogram (**b**) of LSE. The peak numbers, including peaks 1. myricetin-3-galactoside, 2. quercetin-3-glucuronide, 3. isoquercitrin, 4. isoquercitrin, 5. isorhamnetin-3-glucoside, 6. unknown, 7. quercetin, 8. kaempferol, 9. isorhamnetin, and 10. unknown, are referred to in Table 1.

To evaluate the bioactive compounds of lotus seedpod, the LSE was successively extracted with a multistep purification procedure. With this method, the extraction yield of flavonoids from LSE can reach about 85.7% (*w*/*w*) of the aqueous extract. In order to determine the chemical composition of LSE, HPLC/ESI-MS-MS method was performed. As shown in Figure 1a, the HPLC-DAD profiles of LSE monitored at 325 nm were recorded. The UV spectra of all peaks recorded in the chromatograms remarkably revealed most of the peaks with an absorption band at 325 nm and 210–600 nm, characterized as typical of compounds containing a flavonoids moiety [30]. Subsequently, for the identification of the structure, more details on the structural linkage between C-glycosyl and conjugated forms of flavonoids derivatives were obtained by utilizing LC-MS and MS/MS analysis in positive and negative ionization modes (Figure 1b). Further results showed that a total of 10 compounds were identified from LSE, indicating that all of the identified compound classes are belonging to flavonoids derivatives (Table 1). For the first time from the HPLC/ESI-MS-MS method, the unique compositions in LSE are found. As shown in Table 1, quercetin-3-glucuronide (Q3G, 122.44 ± 2.24 mg/100 g dried weight (DW)) was identified to be presented in the highest level in LSE, followed by isorhamnetin-3-glucuronide (30.27 ± 3.46 mg/100 g DW), isorhamnetin-3-glucoside (29.73 ± 4.94 mg/100 g DW), and isoquercitrin (29.44 ± 1.0 mg/100 g DW), as well as only traces of myricetin-3-galactoside (11.52 ± 2.16 mg/100 g DW), kaempferol (2.01 ± 0.61 mg/100 g DW), and quercetin (0.42 ± 0.27 mg/100 g DW) were detected.

### 3.2. LSE Inhibits H_2_O_2_-Mediated Loss of Cell Viability, Inhibition of Insulin Secretion, and Induction of Oxidative Stress in Beta-Cells

**Figure 2:**
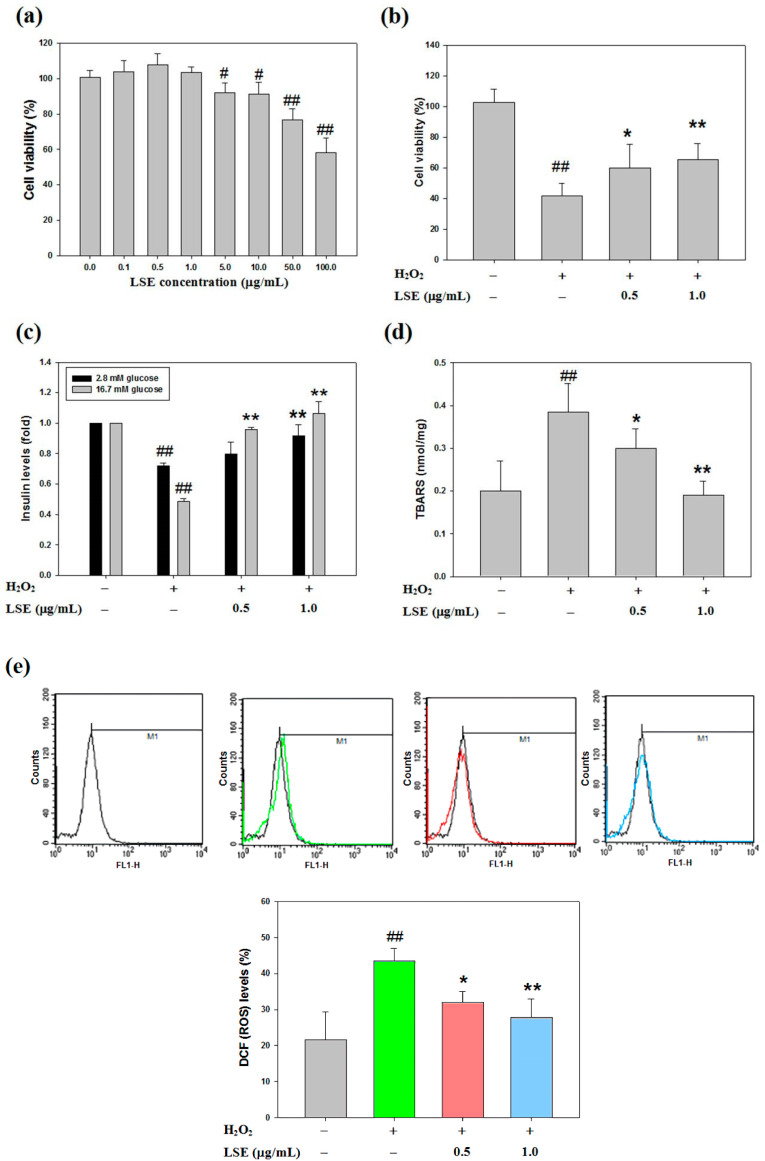
Effects of LSE in combination with H_2_O_2_ on beta-cell viability and oxidative injury. (**a**–**b**) Rat pancreatic beta-cells (RIN-m5F) were treated with various doses of LSE (0–100 μg/mL) alone (**a**) or the indicated doses of LSE (0.5 and 1.0 μg/mL) in combination with 200 μM of H_2_O_2_ (**b**) for 24 h. The cell viability was evaluated by the MTT method. (**c**–**e**) Under the same co-treatment conditions, the insulin secretion (**c**), lipid peroxidation (**d**), and intracellular reactive oxygen species (ROS) level (**e**) were assessed by measuring the glucose-stimulated insulin secretion (GSIS), thiobarbituric acid relative substance (TBARS), and dichlorofluorescein diacetate (DCFH-DA) assay, receptively. The results are represented as mean ± SD (*n* ≥ 3) from three independent experiments. ^#^ *p* < 0.05, ^##^ *p* < 0.01, compared with untreated control; * *p* < 0.05, ** *p* < 0.01, compared with the H_2_O_2_ group.

To explore that LSE is an inhibitor of H_2_O_2_-induced pancreatic beta-cell injury, the effect of LSE on RIN-m5F cell survival by MTT method, indicating the viability, was insignificantly influenced by the treatments of LSE alone at doses between 0.1 and 1.0 μg/mL (Figure 2a). Using an MTT method, a preliminary screening was further performed to determine the effect of LSE at 0.5 or 1.0 μg/mL (Figure 2b) and its main active ingredient in combination with H_2_O_2_ (200 μM) on RIN-m5F cell growth for 24 h. The observation suggested that LSE could dose-dependently attenuate the H_2_O_2_-induced loss of cell viability. Furthermore, the toxic effect of H_2_O_2_ on RIN-m5F cells eventually resulted in impaired insulin secretion, as shown in Figure 2c. To demonstrate whether the H_2_O_2_-impaired GSIS function was restored by LSE, the cells were co-incubated with the different doses (0.5 and 1.0 μg/mL) of LSE plus H_2_O_2_ at 200 μM for 24 h, showing that insulin secretion was upregulated by LSE under H_2_O_2_ stimulation (Figure 2c). In addition, to investigate the degree of cellular oxidative stress elevated by H_2_O_2_ in RIN-m5F cells, the intracellular levels of lipid peroxidation and ROS generation [dichlorofluorescein (DCF) fluorescence] were analyzed (Figure 2d,e). The examination of MDA concentrations revealed that the H_2_O_2_ stimulation led to an increase in lipid peroxidation, compared with the untreated control. Such an increase was concentration-dependently inhibited by the treatments of LSE (Figure 2d). The inhibitory effect of LSE on the ROS production was similar to the result of TBARS under H_2_O_2_ stimulation in the same condition (Figure 2e). According to the above results, Q3G (122.44 ± 2.24 mg/100 g DW) is mainly contained in the composition of the mixture extract LSE (Table 1). The dose of Q3G in LSE at 1.0 μg/mL is approximately 0.12 μg/mL, which is equivalent to 0.25 μM. To examine the protective effect of LSE against H_2_O_2_, the concentration of Q3G at 0.25 μM in the following investigations was used and analyzed. As shown in Figure S2, similar results of cell viability, GSIS, TBARS, and ROS were found in Q3G-treated RIN-m5F cells in the presence of H_2_O_2_.

### 3.3. LSE Inhibits H_2_O_2_-Induced Apoptosis in Beta-Cells

**Figure 3:**
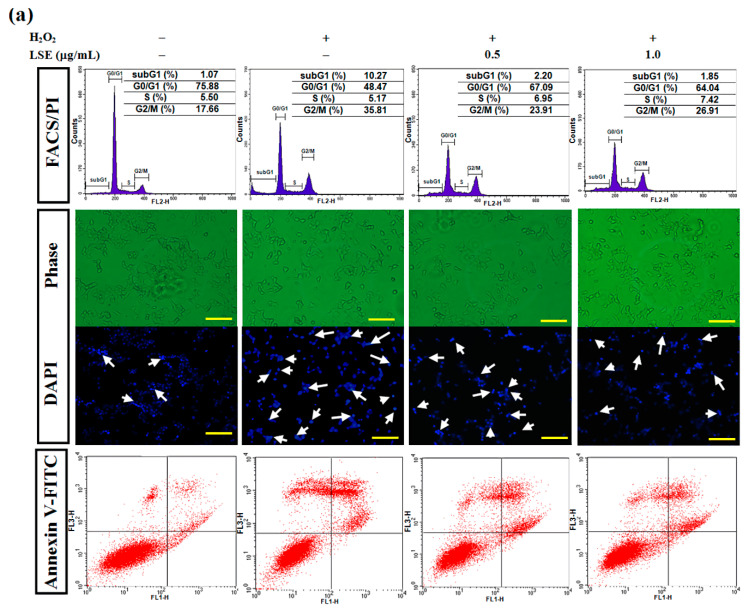

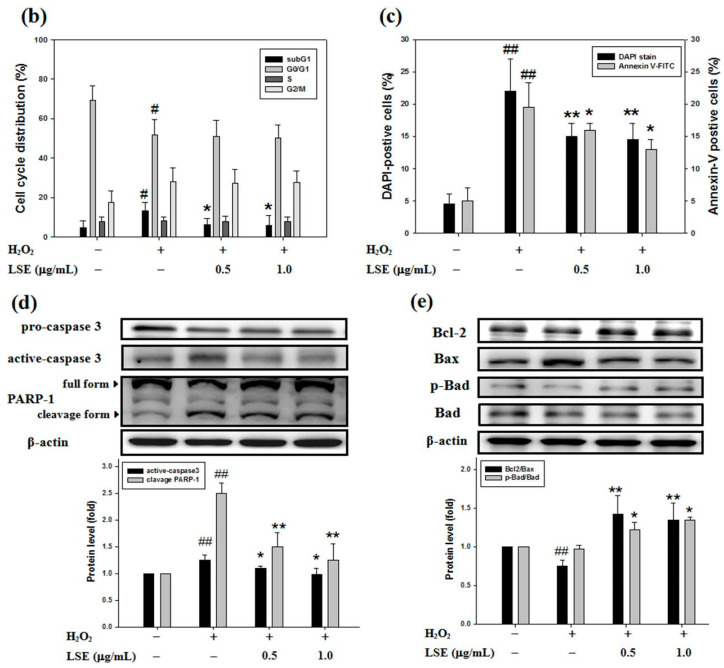
Effect of LSE on H_2_O_2_-induced apoptosis in beta-cells. RIN-m5F cells were treated with or without the indicated doses of LSE (0.5 and 1.0 μg/mL) in the presence of 200 μM of H_2_O_2_ for 24 h. (**a**) The DNA content was analyzed in a flow cytometric method. The position of the subG1 peak, integrated by apoptotic cells, while the peaks in G0/G1, S, and G2/M phases were indicated (*upper panel*). Apoptotic cells were further analyzed by DAPI staining. Arrows indicate apoptotic cells. Panels show phase-contrast microscopy and DAPI staining (*middle panel*). Flow cytometric analysis of cell membranes with annexin V-FITC staining (*lower panel*), a remarkable number of apoptotic cells were stained with positive annexin V-FITC (right quadrant). Images were taken at 200× magnification; scale bar, 30 μm. (**b**) The quantitative assessment of the cell percentage of each phase in the cell cycle was indicated by PI and is represented as the mean ± SD (*n* ≥ 3) from three independent experiments. (**c**) The percentage of DAPI-positive cells relative to total cell number (*left axis*) in each random field (>100 cells) and the proportion of annexin V-positive cells (*right axis*) are, respectively, represented as mean ± SD (*n* ≥ 3) of three independent experiments ± SD. (**d**–**e**) The protein levels of caspase-3 (cysteine-aspartic protease-3), PARP-1 [poly (ADP-ribose) polymerase 1] (**d**), Bcl-2 (B-cell lymphoma 2), Bax (Bcl-2-associated X protein), p-Bad (phospho-Bcl-2-associated death promoter), and Bad (**e**) were assayed by Western blot analysis. β-actin served as an internal control. The quantitative results are presented as the mean ± SD (*n* ≥ 3) from three independent experiments. ^#^ *p* < 0.05, ^##^ *p* < 0.01, compared with untreated control; * *p* < 0.05, ** *p* < 0.01, compared with the H_2_O_2_ group.

Here, to determine whether LSE protected beta-cells against H_2_O_2_-caused injury, a set of classical assays, including FACS/PI, DAPI, and annexin V-FITC methods, was utilized to evaluate cell apoptosis. In a flow cytometric method, the number of hypodiploid cells stained less intensely with PI that can be unequivocally detected from the peak of the cell cycle subG1 phase was determined in RIN-m5F cells exposed to H_2_O_2_ (*upper panel*, Figure 3a). In the 24-h H_2_O_2_-exposed cells, a significant induction in the subG1 phase with fewer cells in the S phase upon H_2_O_2_ alone was observed, compared with untreated control. Furthermore, compared to the H_2_O_2_ alone, the LSE treatments had fewer cell populations in the subG1 phase, indicating that LSE could markedly and dose-dependently lead to the reduction of apoptosis (Figure 3b). The H_2_O_2_-treated cells also represented morphologic changes with apoptotic characteristics, including nuclear condensation and DNA fragmentation. However, LSE alleviated the proportion of apoptosis induced by H_2_O_2_ (*middle panel*, Figure 3a). For the quantification of apoptotic cells, the percentage of DAPI-positive cells showing fragmentation characteristics was approximately elevated by 17.5% in the H_2_O_2_-treated cells. The data in Figure 3c (*left axis*) also indicated that the increase was concentration-dependent, with a 42% reduction, when the H_2_O_2_ model cells were incubated with LSE at 1.0 μg/mL. In order to confirm the above observations, using annexin V-FITC staining, not only early apoptosis and late apoptosis but also necrosis induced by H_2_O_2_ could be quantitatively detected by a FACScan flow cytometer. As shown in Figure 3a (*lower panel*), an induction in apoptotic populations with a minimal effect on cell necrosis in the H_2_O_2_ model cells was confirmed. The adding of LSE to the H_2_O_2_-treated cells showed a substantial reduction of positive annexin V-FITC cell subpopulations (*right axis*, Figure 3c).

The molecular events activating the apoptotic cell death when RIN-m5F cells were incubated with H_2_O_2_ with or without LSE at the indicated doses for 24 h were further studied. Cysteine-aspartic protease (caspase) 3 exists normally as inactive precursors (namely pro-form) with higher molecular weights, 32 kDa. When a cell undergoes apoptosis, caspase-3 is proteolytically activated and cleaved into the lower molecular weights, about 11–20 kDa [27]. As shown in Figure 3d, the changes in protein levels of two key apoptotic markers—active-caspase-3 and its downstream factor, cleavage-form of poly (ADP-ribose) polymerase 1 (PARP-1)—were measured. Western blot analysis showed that levels of active-caspase-3 and cleavage-form of PARP-1 were decreased in the cells co-incubated with H_2_O_2_ and LSE, compared with H_2_O_2_ alone. For the evaluation of Bcl-2 family protein factors, play regulators in apoptotic intrinsic pathway that is upregulated in the prolonged oxidative stress-stimulated beta-cells [5,35], the protein level of anti-apoptotic protein Bcl-2 relative to that of pro-apoptotic protein Bcl-2-associated X protein (Bax), represented as Bcl2/Bax, was decreased after a 24-h incubation of H_2_O_2_, while the reduction was restored by LSE. Significantly, LSE treatments also elevated the phosphorylated level of pro-apoptotic protein Bcl-2-associated death promoter (Bad), representing that LSE let Bad to be inactive (Figure 3e). In addition, Q3G showed the capability to reduce the H_2_O_2_-induced occurrence of apoptosis, similar to the results of LSE (Figure S3).

### 3.4. LSE Activates H_2_O_2_-Induced Autophagy in Beta-Cells

**Figure 4:**
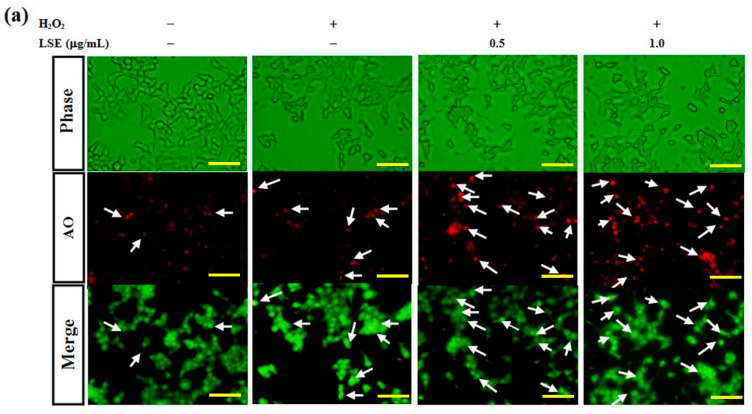

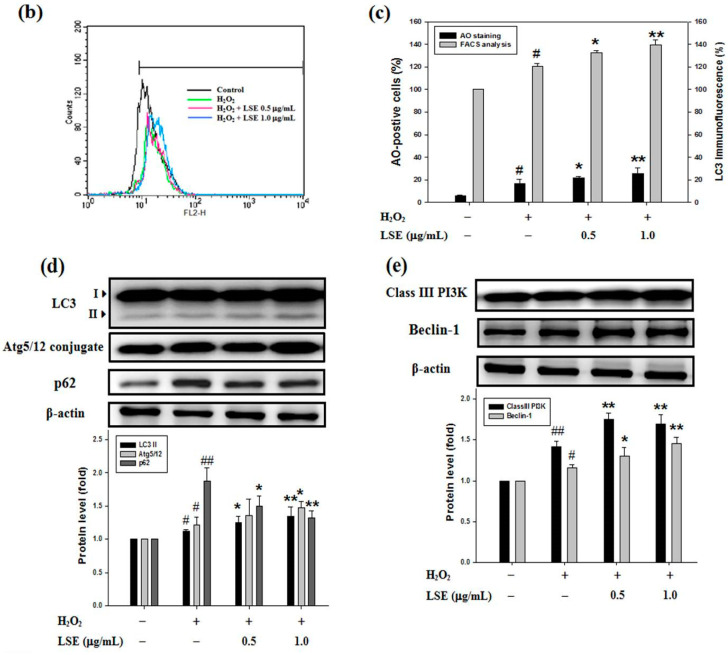
Effect of LSE on H_2_O_2_-mediated autophagy in beta-cells. RIN-m5F cells were treated with or without the indicated doses of LSE (0.5 and 1.0 μg/mL) in the presence of 200 μM of H_2_O_2_ for 24 h. (**a**) Autophagy was analyzed by the acridine orange (AO) stain method. Arrows indicate autophagic cells. Panels represent (from *upper* to *lower*) phase-contrast microscopy (*upper*), AO staining (*middle*), and merge image (*lower*). Images were taken at 200× magnification; scale bar, 30 μm. (**b**) The immunofluorescence intensity of LC3 was carried out by flow cytometry. (**c**) The percentage of AO-positive cells relative to the total cell number (*left axis*) in each random field (>100 cells) and the immunofluorescence intensity of LC3 (*right axis*) are, respectively, shown as mean ± SD (*n* ≥ 3) from three independent experiments. (**d**–**e**) For the analysis of autophagic factors, the protein levels of LC3 (microtubule-associated protein light chain 3) I/II, Atg (autophagy-related genes) 5/12 conjugate, p62 (**d**), class III PI3K (phosphatidylinositol-3 kinase), and Beclin-1 (**e**) were assayed by Western blot analysis. β-actin served as an internal control. The quantitative results are presented as the mean ± SD (*n* ≥ 3) from three independent experiments. ^#^ *p* < 0.05, ^##^ *p* < 0.01, compared with untreated control; * *p* < 0.05, ** *p* < 0.01, compared with the H_2_O_2_ group.

Previous studies have reported that autophagy is crucial for beta-cell survival [10,36]. It was further investigated whether the protective effect of LSE on RIN-m5F cells from H_2_O_2_ oxidative injury was attributed by activating autophagy. To test the hypothesis, the effects of H_2_O_2_ with or without LSE treatments on RIN-m5F cell autophagy were examined. Using AO stain method, untreated control group illustrated slight green fluorescence in whole-cell distribution, while the cells receptively incubated with H_2_O_2_ alone or the combination of H_2_O_2_ and LSE observed the increases in red fluorescent dots in the cytoplasm, showing the formation of acidic autophagolysosomal vacuoles in these treatments (Figure 4a). In order to confirm the synergistic effect of LSE on the H_2_O_2_-induced autophagy, the immunofluorescence intensity signal of LC3, an autophagy indicator, was detected by flow cytometry. Compared with the cells in the H_2_O_2_ group, higher levels of LC3 immunofluorescence intensity were indicated in the cells co-incubated with LSE, as shown in Figure 4b,c (*right axis*), which appeared in a very similar variation tendency to the fluorescence microscopy of AO stain method (*left axis*, Figure 4c).

The action of LSE on LC3 processing and autophagic signal in RIN-m5F cells among different treatments groups were further studied by Western blot analysis. LC3 processing, namely, a rise in the LC3-II expression, was obviously elevated in RIN-m5F cells exposed to H_2_O_2_ alone for 24 h, confirming H_2_O_2_ indeed induce beta-cell autophagy. In the group of LSE plus H_2_O_2_, there were more significant increases in the levels of both LC3-II and Atg5/12 conjugate with a decrease in cellular expression of p62 (Figure 4d), utilized to monitor autophagy flux [36], and in protein levels of class III PI3K and Beclin-1 (Figure 4e). Expectedly, when RIN-m5F cells were stimulated with H_2_O_2_ for 24 h, it was also found that the cellular autophagic events were significantly enhanced after co-treatment with not only LSE but also Q3G (Figure S4).

### 3.5. An Autophagy Inhibitor 3-MA Reduces the Beta-Cell Protective Effect of LSE against Oxidative Injury

**Figure 5:**
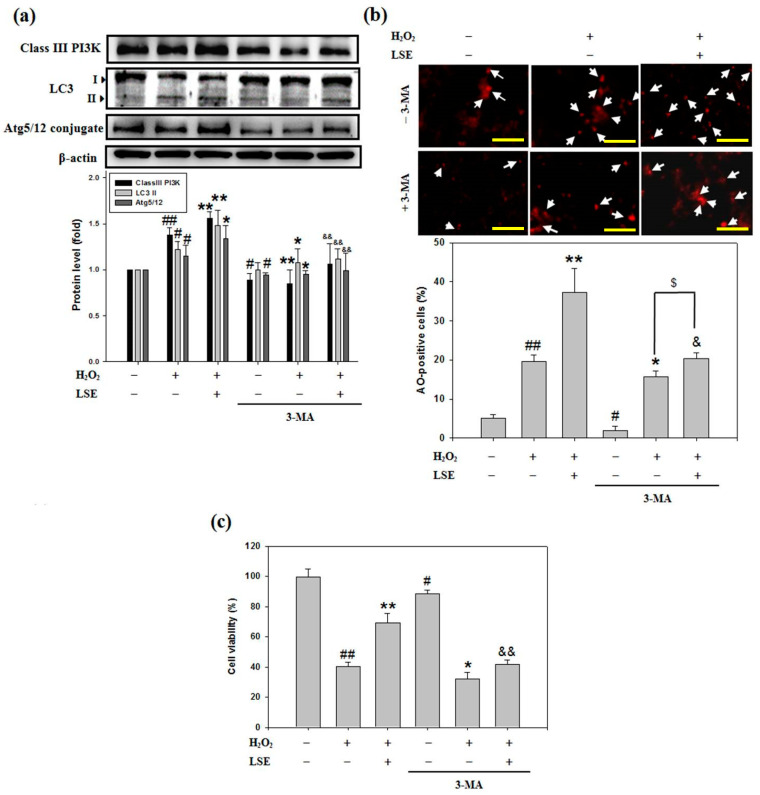
Effect of 3-methyladenine (3-MA) on LSE-induced autophagy and survival in the H_2_O_2_-treated beta-cells. RIN-m5F cells were pretreated with 3-MA (2 mM), then treated with or without LSE (1.0 μg/mL) in the presence of 200 μM of H_2_O_2_ for 24 h. (**a**) The protein levels of class III PI3K, LC3-I/II, and Atg5/12 conjugate were determined by Western blot analysis. β-actin served as an internal control; (**b**) Autophagic cells were analyzed by AO staining. Arrows indicate autophagic cells. Autophagic values were calculated as the percentage of AO-positive cells relative to the total cell number in each random field (>100 cells). Images were taken at 100× magnification; scale bar, 30 μm. (**c**) Cell viability was assayed by MTT method. The quantitative results are presented as mean ± SD (*n* ≥ 3) from three independent experiments. ^#^ *p* < 0.05, ^##^ *p* < 0.01, compared with untreated control (lane 1); * *p* < 0.05, ** *p* < 0.01, compared with group of H_2_O_2_ (lane 2); ^&^ *p* < 0.05, ^&&^ *p* < 0.01, compared with group of H_2_O_2_ plus LSE (lane 3); ^$^ *p* < 0.05, compared with group of H_2_O_2_ plus 3-MA (lane 5).

To explore the mission of autophagy in the beta-cell protective potential of LSE from cellular oxidative injury, whether 3-MA, a specific inhibitor of class III PI3K [37], could interfere with the LSE-mediated cell autophagy and viability was examined. Firstly, when RIN-m5F cells exposed to H_2_O_2_ alone or H_2_O_2_ in combination with LSE, the class III PI3K level (Figure 5a) and acidic autophagolysosome formation (Figure 5b) consistently confirmed the autophagy inhibition by 3-MA. Next, inhibition of class III PI3K significantly decreased the LSE-mediated LC3-II accumulation, Atg5/12 conjugation (Figure 5a), and cell growth (Figure 5c) in the H_2_O_2_-treated cells. As a whole, these data indicated strongly that LSE had a protective effect against the H_2_O_2_-caused beta-cells damage by activating class III PI3K-mediated autophagy. In addition, to study the induction of autophagy flux by LSE, a lysosomal inhibitor CQ at 3 μM was also used during the treatment of LSE to attenuate the degradation of autophagolysosome [38]. The presence of another autophagy inhibitor CQ that disrupts lysosomal function triggered a higher LC3-II protein level in the cells treated with H_2_O_2_ plus LSE than the cells without CQ. The pretreatment of CQ reduced markedly the cell viability of the H_2_O_2_ in combination with LSE treatment group, which coincided well with the data of 3-MA, as evidenced by MTT assay (data not shown), indicating that LSE induced protective autophagy to remove the accumulation of toxic H_2_O_2_ and attenuate RIN-m5F cell injury.

### 3.6. LSE Improves Glucose Intolerance and Insulin Resistance in DM Mice

**Figure 6:**
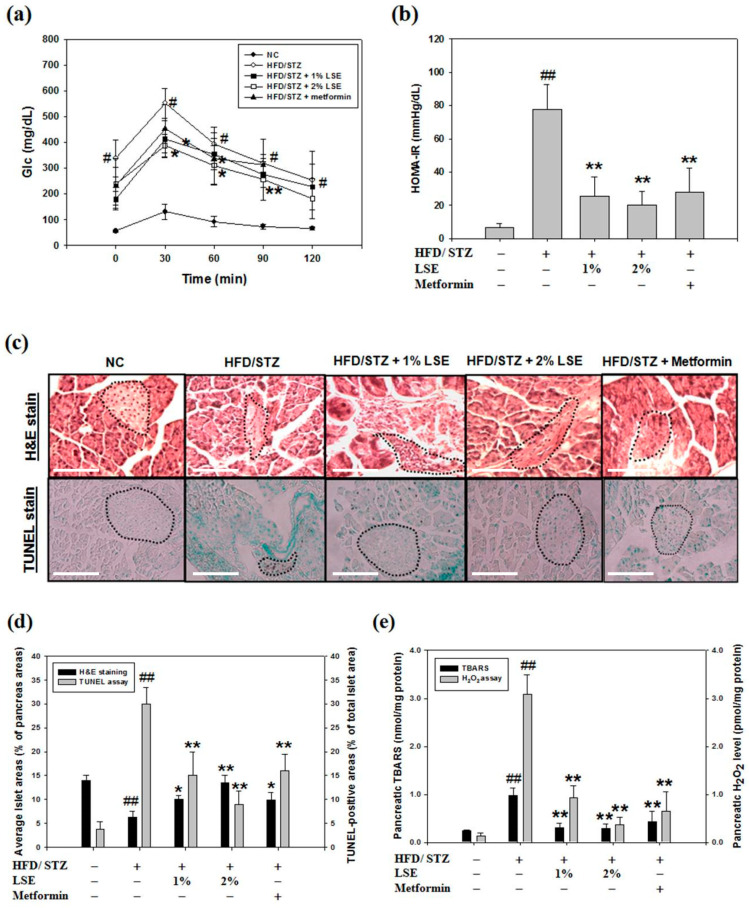

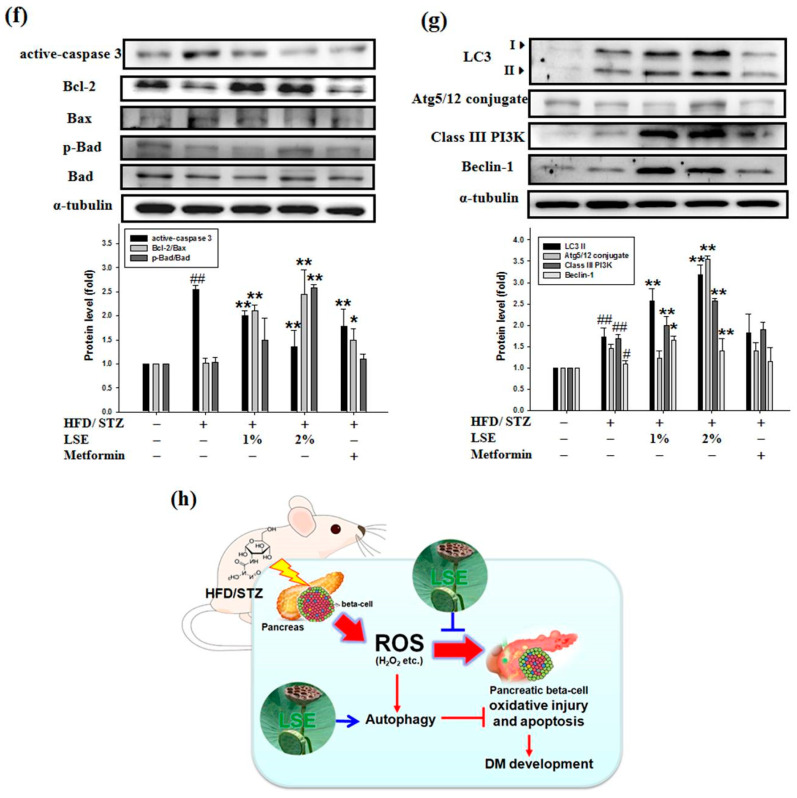
In vivo effect of LSE on diabetes mellitus (DM) symptoms and pancreatic beta-cell dysfunction. BALB/c mice fed on a high-fat diet (HFD) combined with streptozotocin (STZ) injection (HFD/STZ) were randomly divided into four experimental groups. Among groups, two of these groups were fed with LSE at 1% and 2%, whereas the metformin group was administered intragastrically a metformin hydrochloride water solution (300 mg/kg). These mice were sacrificed after 12 weeks. The glucose tolerance (**a**) and insulin resistance (**b**) were, respectively, determined by oral glucose tolerance test (OGTT) and homeostasis model of insulin resistance (HOMA-IR). (**c**) The pancreatic tissues were collected for H&E stain (*upper panel*), and pancreatic islet apoptosis (red-brown color) was analyzed by TUNEL assay (*lower panel*). Images were taken at 200× magnification; scale bar, 50 μm. (**d**) The pancreatic islet areas (*left axis*), relative to the total pancreas, and apoptotic index (*right axis*) were, respectively, measured in the total islet tissues of four groups. Values are expressed as mean ± SD, *n* = 10; (**e**) The intracellular lipid peroxidation (*left axis*) and H_2_O_2_ production (*right axis*) of pancreatic tissues were measured by TBARS and H_2_O_2_ assays, respectively. (**f**,**g**) Western blotting of active-caspase 3, Bcl-2, Bax, p-Bad, Bad (**f**), LC3-I/II, Atg5/12 conjugate, class III PI3K, and Beclin-1 (**g**) protein expressions was performed with the tissue extracts from them. α-tubulin was served as an internal control. The quantitative results are expressed as the mean ± SD (*n* = 10) from one independent experiment. ^#^ *p* < 0.05, ^##^ *p* < 0.01, compared with the negative control (NC); * *p* < 0.05, ** *p* < 0.01, compared with the HFD/STZ group. (**h**) Schematic representation of in vivo beta-cell protective effects of LSE against oxidative injury. HFD/STZ induces intracellular ROS production, leading to pancreatic beta-cell apoptosis and autophagy. While apoptosis contributes to cell injury (by bold arrows), autophagy is induced as a pro-survival mechanism (by regular arrows). LSE performs against ROS via the downregulation of apoptosis and upregulation of autophagy, subsequently mediating the ameliorated effects on pancreatic beta-cell oxidative injury and DM development.

In order to study whether LSE protects beta-cell from oxidative injury in vivo, HFD combined with STZ (HFD/STZ)-induced type 2 DM mouse models treated with LSE (1% or 2%), metformin, or vehicle was set up (Figure S1). Following a 12-week HFD and intraperitoneal injection of STZ, the serum levels of lipid profile, containing total cholesterol (TC) and LDL-cholesterol (LDL-c), and liver and kidney function index, containing alanine transaminase (ALT), blood urea nitrogen (BUN), creatinine (Cre), as well as glucose, were significantly higher in the HFD/STZ group, compared to those in the negative control group; no significant differences were observed in triglycerides (TG), aspartate aminotransferase (AST), and insulin between these two groups (Table S1). The results of glucose tolerance in the HFD/STZ-induced DM mice indicated a noticeable increase in blood glucose levels at all time-points during an OGTT, compared to that of the negative control mice. Oral supplementation of 1% or 2% LSE and for 6 weeks could improve glucose tolerance, as represented in the OGTT results. After six weeks of treatments, the OGTT level of 2% LSE group was markedly lower than that in the metformin group (Figure 6a). Although there were insignificant differences in serum levels of insulin among five groups (Table S1), measurements of glucose and insulin levels were further performed to calculate HOMA-IR to indicate that LSE had an improved effect on insulin sensitivity compared to that of metformin, as shown in Figure 6b. The decrease in the pancreas and bodyweight ratio is a marker of pancreas atrophy and injury. As shown in Table S2, compared to that of the negative control group, the pancreas weight per bodyweight was remarkably decreased in the HFD/STZ group, whereas an increase was observed in the high concentration (2%) of LSE treatment group compared with the HFD/STZ group. On the basis of the hematoxylin and eosin (H&E) stain, the pathological alteration of the pancreas was examined. The data of Figure 6c showed a significant reduction in the areas of the islet and an increase in the number of TUNEL-positive apoptotic cells in the DM model mice when compared to those of the negative normal control. These pathological alterations of the pancreas were rescued by LSE when compared to the DM model mice (Figure 6d), suggesting that LSE exhibited a protective effect on the pancreas. In the metformin group, there were fewer islets areas, but these were rounder shaped than those of LSE groups (Figure 6c,d). These results demonstrated that metformin could repair the cell injury incurred by the pancreatic islets due to induction in HFD/STZ, whereas the groups orally treated with LSE showed better protective effects than metformin in terms of the recovery of HFD/STZ-caused impairment and apoptosis of pancreatic islets (Figure 6d). In addition, the TBARS level in pancreatic tissue was significantly increased in HFD/STZ compared with that in the negative control group, showing the pancreatic oxidative damage. Otherwise, both LSE and metformin reduced the TBARS level (left axis, Figure 6e). In the same limitation, the recovery effects of LSE and metformin on the pancreatic level of H_2_O_2_ were similar to the result of TBARS upon HFD/STZ stimulation (right axis, Figure 6e). Then, Western blotting was used for the examination of apoptotic and autophagic factors in the pancreatic tissue. The levels of apoptotic proteins—active-caspase-3 and Bax—were positively regulated, while the anti-apoptotic proteins—Bcl-2 and p-Bad—were negatively modulated through the HFD/STZ treatment (Figure 6f). Above protein levels were dose-dependently and significantly reversed by LSE and metformin, practically in 2% LSE, as shown in Figure 6f. In order to further study whether the in vivo protective potential of LSE against cellular oxidative injury occurred since it activated autophagy, the changes in protein levels of LC3-II, Atg5/12 conjugate, class III PI3K, and Beclin-1 in the pancreatic tissue were also investigated (Figure 6g). Stimulation with HFD/STZ induced the cellular levels of LC3-II, class III PI3K, and Beclin-1, but not Atg5/12 conjugate, compared to the negative control group. Treatment with LSE at 1% and 2%, not metformin, significantly enhanced these expressions of autophagy proteins in vivo, as shown in Figure 6g.

## 4. Discussion

This is the first report, showing that using HPLC/ESI-MS-MS analysis, LSE contains flavonoids derivatives (Figure 1 and Table 1). Although LSOPC, rich in B-type procyanidins, has been reported to inhibit the AGE formation by scavenging reactive carbonyls and regulating anti-inflammatory signal [17,18], as well as enhance glucose homeostasis in STZ-induced diabetic mice [19], there is a little information as regards the effect of LSE, aqueous extracts of lotus seedpod, on pancreatic beta-cell dysfunction. To our knowledge, this is also the first study, indicating the in vitro and in vivo pancreatic beta-cell protective effect of LSE, rich in C-glycosyl and conjugated forms of flavonoids derivatives.

It is well established that the experiments on diabetic animal models, isolated pancreatic islets, and insulin-releasing cell lines have shown that flavonoids strengthen the insulin secretory capacity and survival processes of beta-cells. The proposed mechanism by which flavonoids preserve beta-cell viability (against glucotoxicity, lipotoxicity, and cytokines) include a decrease of ROS levels, activation of anti-apoptosis pathway, and inhibition of nitric oxide generation [39]. The advantage of flavonoids composition should indispensably contribute to LSE, which has been reported for two main aspects: (1) antioxidation and (2) antidiabetic effects. Among common flavonoids, various studies have indicated that both quercetin and kaempferol exert potential anti-diabetic activities in modulating insulin secretion and managing insulin resistance [40,41]. As shown in Table 1, LSE is mainly composed of Q3G and isoquercetin, which are considered to contribute to their biological properties in addition to quercetin and kaempferol. Previous studies have indicated that Q3G, a major antioxidative quercetin metabolite in human plasma, may exert antioxidant effects on alloxan-induced pancreatic islet damage in zebrafish [42]. Additionally, Q3G is reported to be as effective as quercetin in ameliorating insulin resistance by regulating the insulin receptor substrate 1 (IRS-1) function of the endothelium [43]. Isoquercetin, otherwise known as quercetin-O-beta-glucoside, is supplemented to a high cholesterol diet that could regulate expression and secretion of proprotein convertase subtilisin/kexin type 9 (PCSK9), reversing the hyperlipidemia and hyperinsulinemia caused by the diet [44]. In accordance with the previous and present works, these studies have shown that cooperatively the antioxidant and anti-diabetic effects of LSE may be attributed to their biological activities of the flavonoids components. To date, this present study has further implied that LSE had protective effects against H_2_O_2_-induced beta-cell injury, and this may be performed majorly by Q3G (Figures S2–S4).

Oxidative stress has been involved in various kinds of metabolism disorders, such as DM, NAFLD, hypertension, and cardiovascular disease [45]. Some phytochemicals and antioxidants could ameliorate oxidative stress-induced cytotoxicity and injury. Our recent studies reported that LSE could improve OA-increased oxidative stress and lipotoxicity in hepatocytes [20]. LSE could also inhibit LPS-mediated hepatic inflammation and ROS generation [21]; however, its effect on oxidative stress in beta-cells in response to H_2_O_2_ is still unknown. In this present study, the novel in vitro finding is that a 24-h treatment of LSE dose-dependently protected beta-cells from oxidative injury and dysfunction (Figure 2b–e) to work as an activator to induce autophagy signaling, subsequently attenuating cell death induced by H_2_O_2_ (Figure 3 and Figure 4). A previous study found that the effects of H_2_O_2_ to promote beta-cell viability loss were detectable after 24 h and extended up to 72 h, the longest time researched [22]. Interestingly, however, the actions of a 48-h H_2_O_2_ treatment did not appear to be as strong as those after 24 or 72 h of incubation. The reason for this is not immediately obvious. It has been indicated that H_2_O_2_ activates many elements of the insulin resistance signaling pathway and may also exert dual effects [46]. The molecular mechanisms of H_2_O_2_ are likely complex, and multiple signals may be concerned in the process; thus, these possibilities are currently being studied. As regards LSE, a time-dependent effect of the extract upon H_2_O_2_ administration would be interesting to validate the cytoprotective mechanism and is, thereby, needed to be explored in the future.

Many previous studies have indicated that a high concentration of H_2_O_2_ (more than 100 μM) acts in many cell types, particularly in beta-cells with their high secretory and low antioxidant capacities [22,28], and can mainly lead to apoptosis by directly attacking cellular macromolecules, nucleic acid, and membranes. Previous investigations have shown that LSE is a potent antioxidant agent [20,21]. As shown in Figure 2b,c, LSE had protective effects toward H_2_O_2_-induced cell viability loss and insulin resistance in RIN-m5F cells. Thus, it is likely that, in diabetic animals, LSE acts as an antioxidant to not only scavenge ROS but also protect beta-cells. This point is further supported in data of Figure 2d,e, where LSE markedly suppressed H_2_O_2_-induced lipid peroxidation and ROS production in the cells. The H_2_O_2_-mediated ROS generation has been also thought of as a ubiquitous messenger to modulate autophagic signaling [22,45]. Although it has been accepted that autophagy is a cell death response, many recent reports have explored that it is mostly a cytoprotective program that allows cells to recycle injured organelles and mobilize their energy reserves upon oxidative stress environment [45,47]. Consistent with the past studies, the results of in vitro experiments in this study showed that LSE could protect the beta-cells from H_2_O_2_-induced injury by activating autophagy, which is associated with apoptosis in the cells (Figure 3 and Figure 4). Two autophagy inhibitors—3-MA and CQ—significantly attenuated the protective effects of LSE (Figure 5), demonstrating the favorable action of autophagy in this effect. As commonly known, the class III PI3K combines with Beclin-1 to form a complex, which involves the autophagy initiation through the regulation of other Atgs. Excessive apoptosis was detected in Beclin-1-deprived embryos, implying that Beclin-1 plays an antagonist of apoptosis in embryonic experimental models [36]. It has been reported that, generally, autophagy inhibits apoptotic induction, and caspases activation digests several essential autophagic proteins and shuts off the autophagic mechanism [48]. Among their targets is Beclin-1 or other Atgs, the degradation of which results in the loss of their autophagy-stimulatory function [49]. To end, these results suggest that LSE could enhance the class IIII PI3K/Beclin-1 autophagy signaling regulated by H_2_O_2_, which promotes beta-cell homeostasis. Further works are needed to clarify the interplay between autophagy and apoptosis. Another possibility, which is to be explored, is a protein kinase involved in the protective action of LSE. The candidate protein kinases, such as protein kinase B (PKB), also named Akt, and extracellular signal-regulated protein kinases 1 and 2 (ERK1/2), play important upstream factors of antioxidant proteins, which could protect beta-cells against high ROS-caused oxidative injury by catalyzing the reduction of H_2_O_2_ to nontoxic H_2_O [45]. In light of this, the fact that 3-MA almost completely reversed LSE-suppressed cell death under H_2_O_2_ stimulation and the impairment of autophagy led to the overproduction of cell injury (Figure 5c). Therefore, we cannot rule out it is possible that LSE reduced indirectly beta-cell injury by activating autophagy, in addition to directly quenching ROS through its antioxidant properties, yet future detailed experiments will test this possibility.

The results of the animal experiments also demonstrated that LSE regulated blood lipids, improved the blood glucose, and retained the pancreas weight per bodyweight of the mice, following the feeding of the mice with the combination of HFD and low-dose STZ injection. HFD/STZ has been indicated to induce pancreatic beta-cell apoptosis and a functional defect in insulin sensitivity, and thus, this model was effectively and widely utilized to cause type 2 DM syndrome [50]. STZ is well known to be diabetogenic due to its targeted glucose transporter (GLUT) 2-dependent action in beta-cells; however, the exact mechanism of STZ cytotoxicity is still not clear. Furthermore, both apoptotic and necrotic cell deaths of beta-cells have been reported [51]. Song et al. (2020) found that STZ (35 mg/kg)-injected mice showed near 50% TUNEL-positive cells, served as a hallmark of apoptosis, followed by additional 12-week HFD feeding [52]. The past studies have also demonstrated that a low dose of STZ can acutely induce beta-cell apoptosis as a prediabetic mouse model for the following reasons: (i) mild hyperglycemia; (ii) mild pancreatic islet damage; (iii) apoptosis of beta-cells at <60% [33]. Therefore, the role of antioxidants in DM prevention was able to be investigated. In this study, a model of DM in mice, utilizing an analogous protocol with modification, was successfully established to analyze the therapeutic effect of LSE on glucose intolerance, insulin resistance, and pancreatic cell injury (Figure 6a–c). Consistent with these findings, IHC staining indicated the expression of TUNEL-positive cells in pancreatic islets from pancreases of HFD/STZ-treated mice, as shown in the data of Figure 6c,d. In addition, the activity and toxicity of the extract were further compared with one positive control drug, metformin. As shown in Table S3, in HFD/STZ-induced diabetic mice, we found that the anti-hyperglycemic, glucose tolerance, anti-apoptosis, and autophagy induction effects of LSE were better than metformin, a drug that is widely prescribed to treat diabetes. Moreover, LSE lowered markedly the HOMA-IR values and pancreatic TBARS level, suggesting that LSE induced an insulin-sensitive effect and anti-oxidative stress ability comparable to that of metformin (Table S3). Collectively, in vivo results found that LSE functioned as a potential anti-diabetic agent to protect the pancreas from increasing cell injury, apoptosis, and even DM development.

## 5. Conclusions

The findings of this present study provided evidence suggesting that LSE could protect beta-cells from oxidative injury through the downregulation of apoptosis and upregulation of autophagy (Figure 6h). Especially, these data of in vitro analysis indicated that: (1) LSE reduced the H_2_O_2_ effects on the loss of cell survival and insulin secretion, oxidative stress, and apoptosis of RIN-m5F cells; (2) the beta-cell protective effect of LSE was carried out by activating autophagy; (3) LSE-activated pro-survival autophagy was involved in upregulating class III PI3K/LC3-II signaling. Most significantly, in vivo administration of LSE improved the levels of serum biochemical parameters and DM symptoms, as well as strongly reduced the expressions of apoptosis covering pancreatic tissue from the HFD/STZ-induced DM mice. In summary, the results demonstrated that LSE functions on beta-cells could probably conduce to its protective effects against DM and the related metabolism diseases.

## Figures and Tables

**Table 1:** Identification of the compositions of lotus seedpod aqueous extracts (LSE) by HPLC-DAD (high-performance liquid chromatography-diode array detector), LC-MS (liquid chromatography–mass spectrometry), and LC-MS-MS (liquid chromatography-tandem mass spectrometry) analysis.

Peak No.	Retention Time (min)	Compound	λ_max_ (nm)	[M−H]^−^	MS-MS	Content (mg/100 g DW) ^d^
1	7.97	Myricetin-3-galactoside ^a^	265, 350	479	316	11.52 ± 2.16
2	8.72	Quercetin-3-glucuronide ^a^	224, 254, 354	477	301	122.44 ± 2.24
3	8.89	Isoquercitrin ^a^	265,356	463	301(100), 300(87)	29.44 ± 1.00
4	9.58	Isorhamnetin-3-glucuronide ^a^	268, 355	491	300(100), 301(49), 315(17)	30.27 ± 3.46
5	9.80	Isorhamnetin-3-glucoside ^a^	268, 355	477	314(100), 285(32)	29.73 ± 4.94
6	10.94	Unknown	370	723		13.55 ± 0.07
7	11.38	Quercetin ^b^	254,370	301	151(100), 107(65), 121(49)	0.42 ± 0.27
8	12.21	Kaempferol ^b^	266, 365	285	151(100)	2.01 ± 0.61
9	12.65	Isorhamnetin ^a^	252, 340	315	300(100), 151(19), 107(19)	0.80 ± 0.48
10	15.20	Unknown ^c^	220, 275	311		17.36 ± 0.24
		Total				257.54 ± 15.47

^a^ Compounds were identified in accordance with mass spectra and matched data from literature [34]; ^b^ The identification was further demonstrated and conformed by authentic compound; ^c^ From mass spectra and UV (ultraviolet)-visible absorbance spectra, the compounds were limitedly identified; ^d^ Compounds were quantified from an external calibration of 7-methoxyflavanone (Sigma Chemical Co., St. Louis, MO, USA) in duplicate.

## Supplementary Materials

The following are available online at https://www.mdpi.com/2076-3921/9/8/658/s1, Figure S1: Flow chart of HFD combined STZ treatment-induced DM mice treated with LSE (1% and 2%) or metformin (300 mg/kg) for 6 weeks, Figure S2: Effect of Q3G on H_2_O_2_-induced beta-cell viability loss and oxidative injury, Figure S3: Effect of Q3G on H_2_O_2_-induced beta-cell apoptosis, Figure S4: Effect of Q3G on H_2_O_2_-induced beta-cell autophagy, Table S1: Effect of LSE on the serum biochemical parameters of mice induced by an HFD combined with STZ treatment, Table S2: Effect of LSE on the organ weight/bodyweight ratio of mice induced by an HFD combined with STZ treatment, Table S3: The protective effect(s) of LSE in comparison to the positive control, metformin, in mice induced by an HFD combined with STZ treatment.
