# Supplementary material for: Flavonoids Identification and Pancreatic Beta-Cell Protective Effect of Lotus Seedpod
Source: Antioxidants (Basel). 2020 Jul 24;9(8):658. doi: 10.3390/antiox9080658 (PMC7466071; doi:10.3390/antiox9080658)
Supplement: Supplementary file 1 [file antioxidants-09-00658-s001.pdf]

antioxidants MDPI

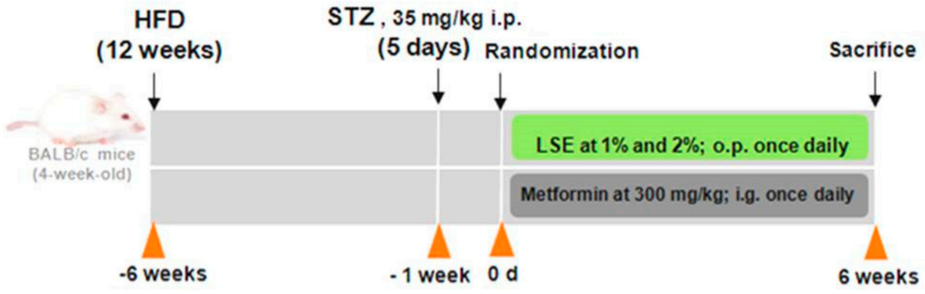

Figure S1. Flow chart of HFD combined STZ treatment-induced DM mice treated with LSE (1% and 2%) or metformin (300 mg/kg) for 6 weeks.

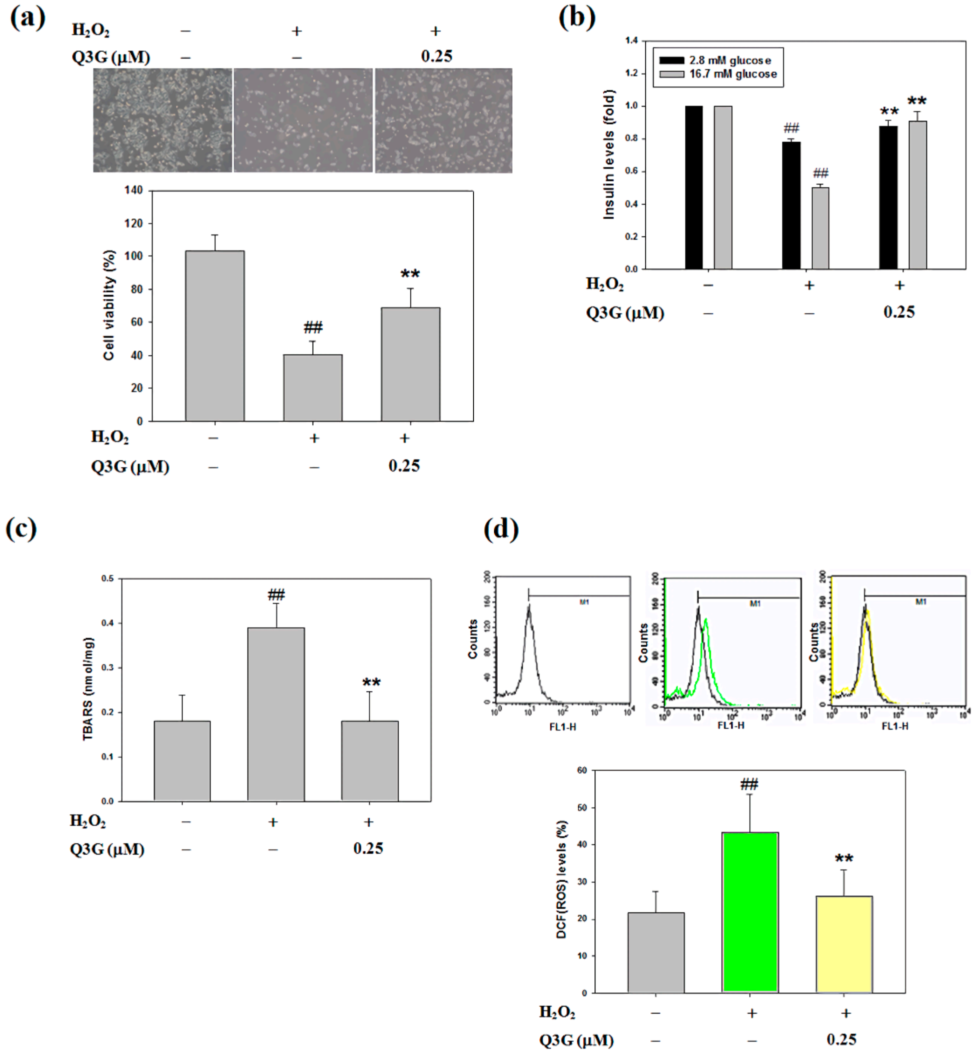

Figure S2. Effect of Q3G on H_2O_2-induced beta-cell viability loss and oxidative injury. **(a)** RIN-5F cells were treated with or without Q3G (0.25 μM) in combination with 200 μM of H_2O_2 for 24 h. Cell viability was assayed by MTT method. **(b-d)** Under the same co-treatment conditions, the intracellular insulin secretion **(b)**, lipid peroxidation **(c)**, and ROS level **(d)** were measured by GSIS, TBARS, and H_2DCF-DA assays, receptively. The results are represented as the mean ± SD (n ≥ 3) from three independent experiments. $^{##}p < 0.01$, compared with the untreated control; $^{**}p < 0.01$, compared with the H_2O_2 group.

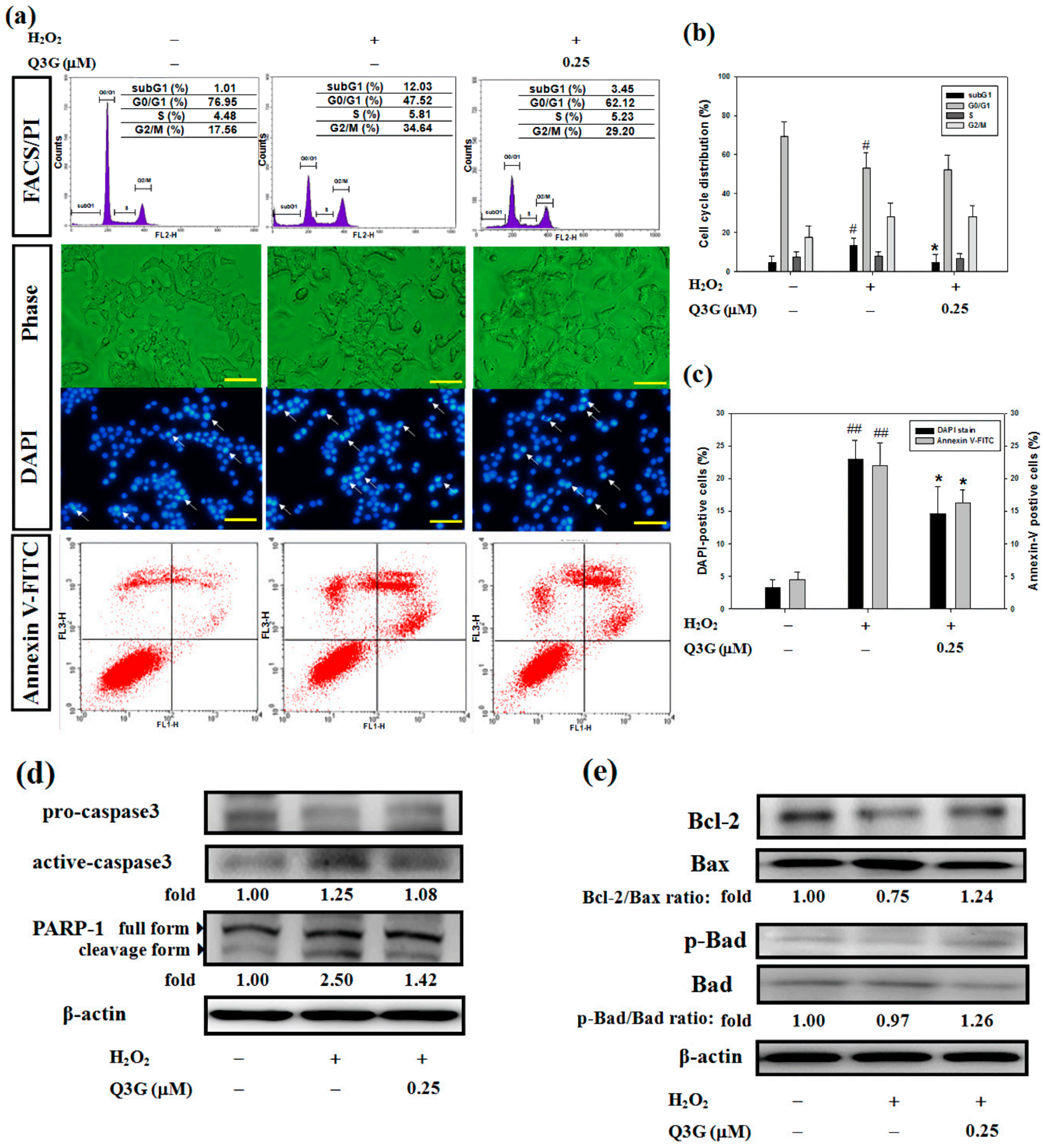

Figure S3. Effect of Q3G on H_2O_2-induced beta-cell apoptosis. RIN-5F cells were treated with or without Q3G (0.25 μM) in the presence of 200 μM of H_2O_2 for 24 h. **(a)** The DNA content was assayed by flow cytometry. The position of the subG1 peak, integrated by apoptotic cells, and the peaks of G0/G1, S, and G2/M phases are indicated (*upper panel*). Apoptotic cells were further detected by DAPI method. Arrows indicate apoptotic cells. Panels show phase-contrast microscopy and DAPI staining (*middle panel*). Flow cytometry method of cell membranes with annexin V-FITC staining (*lower panel*), a significant number of apoptotic cells were stained with positive annexin V-FITC (right quadrant). **(b)** Quantitative assessment of the cell percentage in each phase of the cell cycle was indicated by PI and is represented as the mean ± SD (n ≥ 3) from three independent experiments. **(c)** The percentage of DAPI-positive cells (*left axis*) relative to the total cell number in each random field (> 100 cells) and the proportion of annexin V-positive cells (*right axis*) are, respectively, represented as mean ± SD (n ≥ 3) from three independent experiments ± SD. $^{\#}p < 0.05$, $^{\#\#}p < 0.01$, compared with the untreated control; $^{*}p < 0.05$, compared with the H_2O_2 group. **(d-e)** The protein levels of caspase-3, PARP-1 **(d)**, Bcl-2, Bax, p-Bad, and Bad **(e)** were analyzed by Western blot analysis. β-actin served as an internal control. Determined expressions of the protein were quantified by densitometric analysis with that of control being 1.00 fold, as shown just below the gel data.

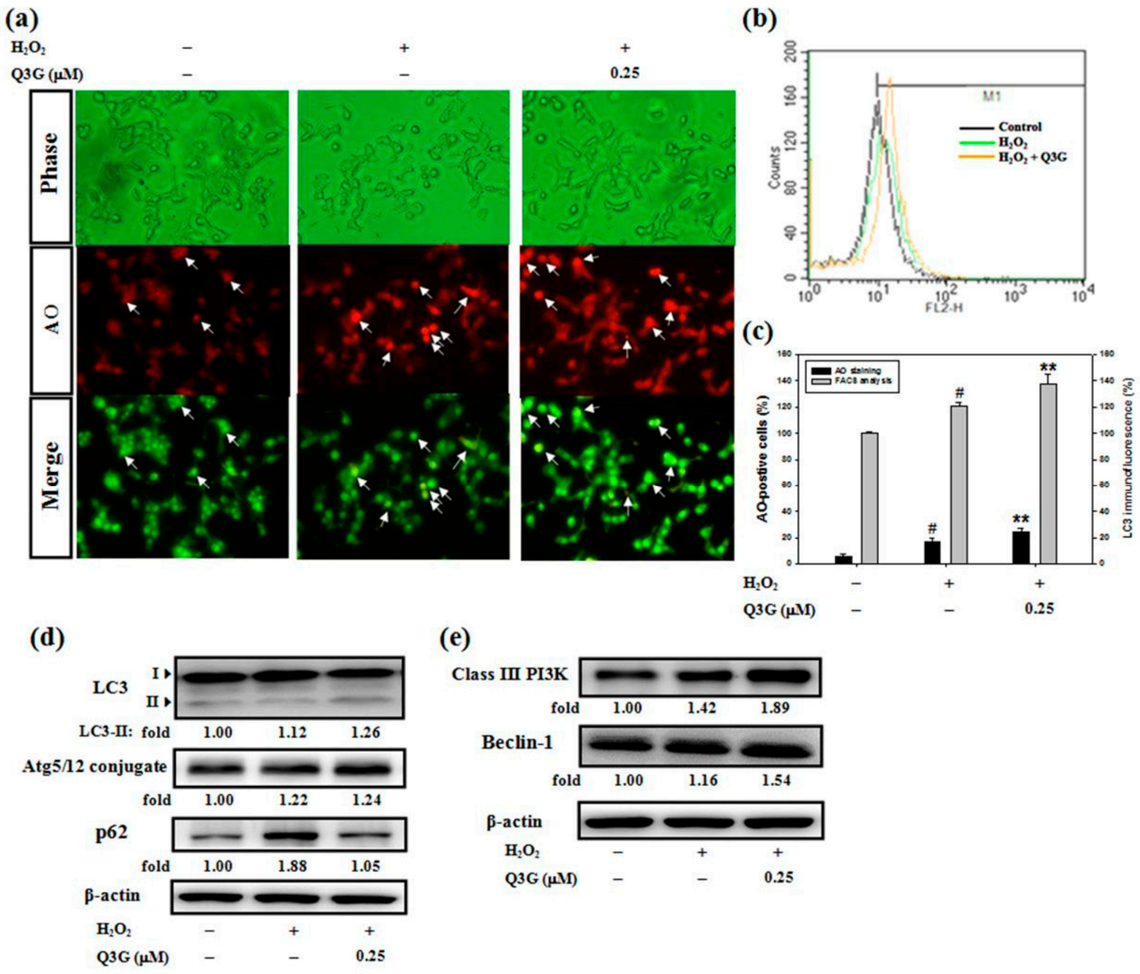

Figure 4. Effect of Q3G on H_2O_2-induced beta-cell autophagy. RIN-5F cells were treated with or without Q3G (0.25 μM) in the presence of 200 μM of H_2O_2 for 24 h. **(a)** Autophagy was assayed by the AO method. Arrows indicate autophagic cells. Panels show (from *upper* to *lower*) phase-contrast microscopy (*upper*), AO staining (*middle*), and merge image (*lower*). **(b)** The relative level of LC3 immunofluorescence intensity was carried out by flow cytometry. **(c)** The percentage of AO-positive cells relative to the total cell number (*left axis*) in each random field (> 100 cells) and the immunofluorescence intensity of LC3 (*right axis*) are, respectively, represented as mean ± SD (n ≥ 3) from three independent experiments. $^{\#}p < 0.05$, compared with the untreated control; $^{**}p < 0.01$, compared with the H_2O_2 group. **(d-e)** The protein levels of LC3I/II, Atg5/12 conjugate, p62 **(d)**, class III PI3K, and Beclin-1 **(e)** were assayed by Western blot analysis. β-actin served as an internal control. Determined expressions of the protein were quantified by densitometric analysis with that of control being 1.00 fold, as shown just below the gel data.

Table 1. Effect of LSE on the serum biochemical parameters of mice induced by an HFD combined with STZ treatment. [a]

Groups Variable [b]	Control	HFD/STZ	HFD/STZ + 1% LSE	HFD/STZ + 2% LSE	HFD/STZ + Metformin
TG (mg/dL)	152.67 ± 18.88	141.67 ± 20.98	106.50 ± 19.36 [e]	93.50 ± 21.46 [f]	103.73 ± 25.22
TC (mg/dL)	165.60 ± 17.44	223.75 ± 21.42 [c]	227.33 ± 8.14	182.75 ± 17.06 [e]	195.27 ± 8.26[e]
LDL-c (mg/dL)	35.50 ± 6.35	67.25 ± 8.81 [c]	66.67 ± 12.06	59.75 ± 5.91 [e]	61.08 ± 10.28
HDL-c (mg/dL)	89.20 ± 5.45	125.00 ± 17.36 [c]	118.00 ± 14.18	142.50 ± 8.06	115.24 ± 13.25
LDL-c/HDL-c	0.41 ± 0.08	0.53 ± 0.02 [d]	0.56 ± 0.09	0.42 ± 0.07 [e]	0.54 ± 0.06
AST (U/L)	226.00 ± 56.20	250.00 ± 101.08	228.33 ± 43.02	213.50 ± 33.26	202.58 ± 30.61
ALT (U/L)	76.00 ± 9.90	99.50 ± 9.88 [d]	119.00 ± 36.77	82.00 ± 32.49	68.34 ± 16.08 [e]
BUN (mg/dL)	21.20 ± 2.52	34.04 ± 6.86 [c]	14.67 ± 1.39 [f]	16.80 ± 2.81 [f]	15.68 ± 2.56 [f]
Cre (mg/dL)	0.38 ± 0.05	0.50 ± 0.07 [d]	0.50 ± 0.08	0.46 ± 0.11	0.45 ± 0.07
Glc (mg/dL)	50.10 ± 7.14	342.83 ± 37.30 [c]	225.00 ± 99.82 [e]	182.00 ± 81.43 [f]	192.04 ± 75.31 [f]
Insulin (mg/dL)	1.74 ± 0.46	1.76 ± 0.45	1.70 ± 0.33	1.77 ± 0.34	2.05 ± 0.56

[a] Values were represented as the mean ± SD (n = 10/group). Duration of the experiment = 6 weeks. The results were statistically assayed with Student's t-test. [b] TG, triglycerides; TC, total cholesterol; LDL, low-density lipoprotein; HDL, high-density lipoprotein; AST, aspartate aminotransferase; ALT, alanine transaminase; BUN, blood urea nitrogen; Cre, creatinine. [c] $p < 0.01$, [d] $p < 0.05$, compared with the negative control group. [e] $p < 0.05$, [f] $p < 0.01$, compared with the HFD/STZ group.

Table 2. Effect of LSE on the organ weight/bodyweight ratio of mice induced by an HFD combined with STZ treatment.[a]

Groups Organ Weight (mg)/Body Weight (g) Ratio	Control	HFD/STZ	HFD/STZ + 1% LSE	HFD/STZ + 2% LSE	HFD/STZ + Metformin
Pancreas	3.39 ± 1.15	1.39 ± 0.27 [b]	1.93 ± 0.56	2.54 ± 1.20 [c]	2.23 ± 1.27
Liver	46.32 ± 2.98	68.10 ± 5.56 [b]	63.81 ± 5.31	58.12 ± 3.08 [c]	61.25 ± 6.62
Kidney	18.77 ± 1.25	27.91 ± 1.67 [b]	27.88 ± 1.06	27.43 ± 1.16	28.16 ± 2.08

[a] Values were represented as the mean ± SD (n = 10/group). Duration of the experiment = 6 weeks. The results were statistically assayed with Student's *t*-test. [b] $p < 0.01$, compared with the negative control group. [c] $p < 0.05$, compared with the HFD/STZ group.

Table 3. The protective effect(s) of LSE in comparison to the positive control, metformin, in mice induced by an HFD combined with STZ treatment.

Treatments [a]	Diabetes Symptoms		Cell Injury		Apoptosis		Autophagy
	OGTT peaked at 30 min	HOMA-IR	Islet areas	TBARS	TUNEL+ areas	Active-casp 3 expression	LC3-II expression
**LSE**	38.95% (↓) [b]	80.87% (↓)	93.57% (↑) [c]	92.00% (↓)	80.15% (↓)	76.77% (↓)	195.95% (↑)
**Metformin**	15.91% (↓)	70.18% (↓)	46.53% (↑)	75.67% (↓)	53.44% (↓)	49.68% (↓)	12.33% (↑) [d]

[a] In the mice after induction of diabetes, followed by treatment with LSE (2%) or metformin (300 mg/kg), serum biochemical parameters, histological alterations, apoptosis, and autophagy marker were analyzed, as described in Materials and Methods. Duration of the experiment = 6 weeks. [b] Values were represented as inhibition (↓) rate of OGTT peaked at 30 min; HOMA-IR, TBARS, $TUNEL^+$ areas, and active-caspase 3 expressions were calculated in LSE or metformin group compared with the HFD/STZ group, respectively. [c] Values were represented as induction (↑) rate of islet areas, and the LC3-II expression was calculated in LSE or metformin group compared with the HFD/STZ group, respectively. [d] No significant effect on LC3-II expression was observed in the metformin group compared with the HFD/STZ group.
